# Rapid generation of antigen-specific monoclonal antibodies from single mouse B cells

**DOI:** 10.52601/bpr.2025.240067

**Published:** 2025-08-31

**Authors:** Xuanxiu Ren, Yiwei Zhang, Gan Zhang, Shangyu Yang, Feiyang Yu, Rao Cheng, Zengqin Deng, Haiyan Zhao

**Affiliations:** 1 State Key Laboratory of Virology and Biosafety, College of Life Sciences, Wuhan University, Wuhan 430072, China; 2 State Key Laboratory of Virology and Biosafety, Wuhan Institute of Virology, Chinese Academy of Sciences, Wuhan 430207, China; 3 Hubei Jiangxia Laboratory, Wuhan 430207, China

**Keywords:** Monoclonal antibodies, Mouse antibody, Single B cell PCR, High-throughput antibody expression

## Abstract

Identifying immunoglobulin (Ig) genes from antigen-specific B cells is crucial for understanding immune responses and generating monoclonal antibodies for diagnostic and therapeutic purposes. Despite single B cell PCR-based mouse antibody development is well established, several practical challenges remain. Here, we present an optimized protocol for the sequencing and cloning of variable regions of antibodies from single antigen-specific mouse B cells, along with high-throughput antibody expression and characterization. This method builds upon existing techniques, incorporating laboratory refinements and detailed troubleshooting insights. By integrating fluorescence-activated cell sorting (FACS) with reverse transcription polymerase chain reaction (RT-PCR) to amplify immunoglobulin heavy and light chain genes, along with a 12-well format for antibody expression, our refined approach enables efficient monoclonal antibody production and functional screening, thereby accelerating the antibody discovery workflow across a range of experimental applications.

## INTRODUCTION

Antibodies, naturally produced by B lymphocytes (B cells), are critical components of humoral immune responses and play key roles in both diagnostics and therapeutics. Characterizing the antibody response at the monoclonal antibody (mAb) level is essential for understanding B-cell-mediated humoral immunity and advancing vaccine development. Recent advancements in mAb generation have revolutionized several fields in biology and medicine, particularly cancer immunotherapy, infectious disease management, and autoimmune disorder treatment (Lu *et al*. 2020; Taylor *et al*. 2024). The remarkable clinical outcomes associated with mAb therapies highlight the pressing need for robust and efficient antibody development methodologies, which continue to be a highly important area of scientific research (Chan and Carter 2010; Mullard 2021; Pantaleo *et al*. 2022; Zinn *et al*. 2023).

Hybridoma technology and single B cell cloning are two of the most commonly used approaches for generating mouse antibodies (Cotton and Milstein 1973; Walker and Burton 2018). While hybridoma technology remains widely used for mAb production, it is inherently labor-intensive and time-consuming, often resulting in variable success rates and low yield (Köhler and Milstein 1975; Bradbury *et al*. 2011; Lu *et al*. 2020). By contrast, single B cell sorting, combined with single-cell PCR, enables the direct isolation of antigen-specific B cells and the amplification of immunoglobulin variable region genes, offering a more streamlined path toward mAb production (Tiller 2011). Unlike the hybridoma method, B cell sorting is faster, cost-effective, and yields highly specific monoclonal antibodies without requiring time-intensive screening and selection steps.

Several protocols for single-cell PCR-based mouse antibody cloning have been reported, providing valuable insights for antibody development (Tiller *et al*. 2009; von Boehmer *et al*. 2016; Ho *et al*. 2016). However, practical challenges such as low cloning efficiency and difficulties in obtaining complete variable region sequences hinder the widespread application of this method. To address these issues, we have optimized a protocol for antibody development from single antigen-specific B cells in mice. This protocol incorporates multiple laboratory refinements and troubleshooting strategies aimed at maximizing amplification efficiency and streamlining the cloning workflow (Tiller *et al*. 2009; von Boehmer *et al*. 2016). Importantly, we also include antigen-specific B cell induction, single B cell sorting strategies, and high-throughput antibody expression-key steps that are often missing in most published protocols.

**Figure 1:**
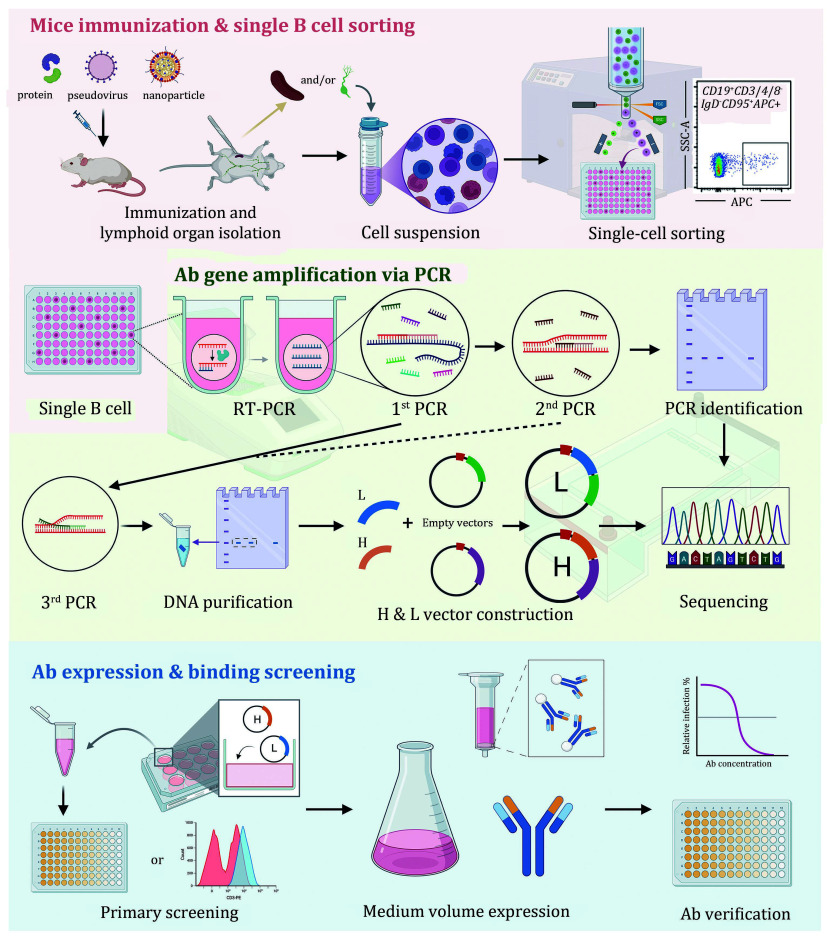
A flowchart of rapid generation of antigen-specific monoclonal antibodies from single mouse B cells. The procedure consists of three main steps: mice immunization and single B cell sorting (with a pink background), antibody (Ab) gene amplification via PCR (with a light green background), and Ab expression and binding screening (with a light blue background)

In summary, we present a comprehensive experimental protocol for efficient antibody generation and characterization. This includes antigen-specific B cell induction, single B cell sorting and cloning, construction of mAb expression vectors, and a high-throughput 12-well plate system for mAb expression and functional screening (Fig. 1). This protocol provides a robust framework for antibody discovery, ultimately contributing to the advancement of monoclonal antibody development in diverse experimental applications.

## MATERIALS AND EQUIPMENT

### Reagents

• 1× Phosphate Buffered Saline (PBS), Servicebio, Cat. #G4202

• Fetal Bovine Serum (FBS), ExCell Bio, Cat. #FSP500

• AddaVax, InvivoGen, Cat. #vac-adx-10

• 0.5 mol/L EDTA, pH 8.0, Biosharp, Cat. #BL518A

• RNasin Plus RNase Inhibitor, Promega, Cat. #N2615

• Fixable viability stain 780, BD Biosciences, Cat. #565388

• Ms CD3e BV510, BD Biosciences, Cat. #563024

• Ms CD4 BV510, BD Biosciences, Cat. #563106

• Ms CD8a BV510, BD Biosciences, Cat. #563068

• Ms CD19 PE-Cy7, BD Biosciences, Cat. #552854

• Ms IgD PerCp-Cy5.5, BD Biosciences, Cat. #564273

• Ms CD95(Fas) PE, BD Biosciences, Cat. #554258

• Streptavidin-APC, BD Biosciences, Cat. #554067

• 10× PBS, Servicebio, Cat. #G4207

• EZ-Link NHS-PEG4-Biotin, ThermoFisher, Cat. #A39259

• Phanta Max Super-Fidelity DNA Polymerase, with 10× DNA Loading buffer, Vazyme, Cat. #P505

• HiScriptII 1st Strand cDNA Synthesis Kit, Vazyme, Cat. #R211

• 2× Es Taq MasterMix (Dye), CWBIO, Cat. #CW0690S

• ClonExpress Ⅱ One Step Cloning Kit, (Vazyme, Cat. #C112

• Agarose, Biosharp, Cat. #BS081

• GimiRun DM5000 DNA Marker, Gimmico, Cat. #GM6055

• Sodium chloride (NaCl), Sinopharm, Cat. #10019318

• Oxoid™ Tryptone, ThermoFisher, Cat. #LP0042B

• Oxoid™ Yeast Extract Powder, ThermoFisher, Cat. #LP0021B

• Polyethylenimine (PEI), Polysciences, Cat. #24765 (Caution: Irritant, handle in a hood with gloves)

• rProtein A Beads 4FF (for antibody purification), Smart Lifesciences, Cat. #SA015100

• SMM 293-TII Expression medium (for cell culture), SinoBiological, Cat. #M293TII

• RPMI 1640 Medium, Monad, Cat. #CC00201S

• Penicillin-Streptomycin Solution (antibiotic), BasalMedia, Cat. #S110JV

• Erythrocytes Lysate, Biosharp, Cat. #BL503B

### Equipment

• Dissection scissors and forceps, sterilized

• 50 mL centrifuge tubes, Labselect, Cat. #CT-002-50A

• 15 mL centrifuge tubes, Labselect, Cat. #CT-002-15A

• 70 µm cell Strainer, Biologix, Cat. #15-1070

• 5 mL round-bottom tube without cell-strainer cap, Falcon, Cat. #352052

• 96-well mini skirt PCR plate, Bio-Rad, Cat. #HSP9601

• Cover foil for sorting plate, Bio-Rad, Cat. #MSF1001

• Cover foil for PCR plate, Biosharp, Cat. #BS-SF-A

• E-Z 96 Endo-Free Plasmid Kit, Omega, Cat. #D1099-01

• Affinity chromatography column, Sangon Biotech, Cat. #F506607-0001

• Amicon Ultra-15 Centrifugal Filter Unit, Merck Millipore, Cat. #UFC801024

• BD FACSAria III flow cytometer (for cell sorting), BD Biosciences

• NanoDrop 2000/2000c Spectrophotometer, ThermoFisher, Cat. #ND-2000

### Reagent setup

• FACS Buffer: Add 10 mL FBS and 1 mL 0.5 mol/L EDTA (pH 8.0) to 490 mL 1× PBS. Store at 4°C for up to one month.

• RT-PCR Catch Buffer for 96-Well Plate: For one plate, combine 18 µL RNasin Plus RNase Inhibitor, 7.2 µL 1 mol/L Tris (pH 8.0), and 694.8 µL nuclease-free water for a total volume of 720 µL.

• Ampicillin Stock Solution (100 mg/mL): Dissolve 1 g ampicillin sodium salt in 10 mL ddH₂O, then filter through a 0.22-µm membrane for sterilization. Store at −20°C.

• LB-Ampicillin Agar Plates: Add 10 g tryptone, 5 g yeast extract powder, 10 g sodium chloride, and 15 g agar to 1 L ddH₂O. Autoclave the solution. Once the LB agar has cooled to 55°C, add 100 mg ampicillin, then pour it into Petri dishes. Store at 4°C for up to one month.

• Agarose Gel (1% *w*/*v*): Dissolve 1 g agarose in 100 mL 1× TAE and heat until dissolved. Once the solution has cooled to 55°C, add 5 µL Gel Red, then pour into the gel tray.

## PROCEDURES

### Step 1: Mice immunization and single B cell sorting

#### Step 1.1: Antigen-specific B cell induction and single-cell suspension from spleen and lymph nodes

Step 1.1.1: To generate functional antibodies, we usually immunize BALB/c or C57BL/6 mice with purified protein. A standard protocol involves intramuscular (i.m.) administration of three doses of 10 µg recombinant protein emulsified with the adjuvant AddaVax at three-week intervals.

**[Tip]** We have tested recombinant proteins, pseudoviruses, and nanoparticles. We found that pseudovirus and nanoparticle immunogens can induce higher antibody titers and broader epitope coverage compared with recombinant proteins (Ren *et al*. 2024; Zhou *et al*. 2024).

Step 1.1.2: Before each immunization, collect blood samples and measure antigen-specific antibody titers in mouse serum.

Step 1.1.3: When antibody titers reach a high level (typically indicated by an enzyme-linked immunosorbent assay [ELISA] binding signal of above 1.5 at a 1:10,000 serum dilution), administer a final booster dose of 25 µg homologous protein. The boost antigen is selected based on experimental purpose and is intended to recall memory B cells, thereby enhancing the antibody responses induced by the prior immunogens. The booster antigen is divided into two equal doses and administered via intraperitoneal (i.p.) and intravenous (i.v.) routes.

**[Tip]** Optimizing the antigen dose and immunization schedule is essential to achieving a robust immune response and high antibody titers (Wang *et al*. 2023; Liang *et al*. 2024; Schunk and Macallum 2005; Pollard and Bijker 2021).

Step 1.1.4: Mice are euthanized five to seven days after the final booster to collect immune cells at peak activity for subsequent analysis.

Step 1.1.5: After euthanizing the mouse, harvest the spleen or lymph nodes to make a single-cell suspension. Place the spleen or lymph nodes on a 70-µm cell strainer in a dish with RPMI 1640 medium supplemented with 2% FBS.

**[Tip]** To ensure cell viability, related handling should be performed on ice or at 4°C whenever possible. While the spleen is frequently used for B cell isolation, lymph node cells are also valuable sources for sorting.

Step 1.1.6: Grind the spleen using the plunger of a 2 mL syringe on a cell strainer until no visible tissue pieces remain, allowing the cells to be released into the medium.

Step 1.1.7: Collect the suspension from Step 1.1.6 and centrifuge at 500*g* for 4 min.

Step 1.1.8: Discard the supernatant and resuspend the cell pellet with 3 mL of erythrocytes Lysate for 3 min at room temperature to lyse red blood cells.

**[Tip]** Carefully control the lysis time to minimize any impact on cell viability.

Step 1.1.9: Wash the cells by adding 7 mL RPMI 1640 (2% FBS), centrifuge again at 500*g* for 4 min, and discard the supernatant.

Step 1.1.10: Resuspend the cells in 500 µL RPMI 1640 for cell counting. In our laboratory, cell counting is typically performed using a hemocytometer. A 10 µL of the cell suspension is first diluted 50-fold, after which 10 µL of the diluted suspension is mixed with 10 µL of trypan blue. Then, 10 µL of this mixture is loaded onto the hemocytometer. The total number of viable cells counted in the 16 squares at the four corners is recorded as ‘*n*’. The concentration of live cells in the original suspension is then calculated using the formula: *n*/4 × 10^4^ × 100 (dilution factor).

**[Tip]** B cells and T cells represent a large proportion of total spleen cells, and their size (8−10 μm) is smaller than that of commonly maintained laboratory cells (*e*.*g*., Vero E6 and 293T). Be sure to adjust the parameters when using the automated cell counter to ensure accurate cell counting.

#### Step 1.2: Single B cell sorting by flow cytometry

Step 1.2.1: Prepare at least 3−10 million splenocytes suspended in 100 µL pre-chilled staining buffer (2% FBS, 1 mmol/L EDTA in PBS buffer).

Step 1.2.2: Prepare control tubes for compensation calculation by using 0.5 million cells for each fluorescent antibody.

Step 1.2.3: Add biotinylated antigen to the sample cells.

**[Tip]** If the biotinylated antigen is being used for the first time, optimizing staining concentrations is recommended.

Step 1.2.4: Incubate the cells with the antigen at 4°C for 30 min.

Step 1.2.5: Wash the cells twice with staining buffer and centrifuge at 500*g* for 4 min after each wash.

Step 1.2.6: Add a mix of fluorescent antibodies such as CD19 PE-Cy7, CD95 PE-A, and Streptavidin-APC to the sample. Incubate at 4°C for 30 min in the dark.

Step 1.2.7: Set up the flow cytometer by running the blank and single-color control tubes first to set compensation. Adjust the voltage as needed to ensure signal detection.

Step 1.2.8: Gate for live B cells based on markers: 780^−^ CD19^+^ CD3/4/8^−^ IgD^−^ CD95^+ ^APC^+ ^for antibody-secreting B cells, and 780^−^ CD19^+^ CD3/4/8^−^ IgD^−^ CD95^−^ APC^+^ for memory B cells.

**[Tip]** Based on our laboratory experiences, single-cell PCR efficiency is much higher for antibody-secreting B cells than memory B cells at the time we collect the peripheral immune organs. However, sorting memory B cells remains a valuable option in certain contexts.

Step 1.2.9: Sort these cells into a 96-well plate containing 7 µL RNase-free catch buffer per well, and immediately freeze the plate at −80°C or on dry ice for subsequent steps. It is recommended to leave the last two wells unsorted, as they will be reserved for negative and positive controls in the following steps.

**[Tip]** Consider exploring various staining strategies, such as double immunogen staining or direct IgG staining. However, it is crucial to establish a clear strategy and ensure that different staining markers do not interfere with each other. Additionally, proper compensation calculation is essential, which relies on the quality of the single-color stained control samples. To improve gating accuracy, cells can be treated with a specific purpose. For example, to differentiate live and dead cells, a portion of the sample can be heated to 65°C prior to mixing with the live cells, followed by the application of fixable viability stains.

### Step 2: Antibody gene amplification via PCR

#### Step 2.1: Reverse transcription (RT) for cDNA synthesis

Step 2.1.1: Remove the sorted 96-well plate from −80°C storage and place it on ice for 5 min.

**[Tip]** The 96-well plate containing sorted single cells from the above steps is used here for reverse transcription; therefore, it is essential to consider the maximum and minimum temperature tolerances of the plate and its sealing film.

Step 2.1.2: Centrifuge the plate at 400*g* for 30 s at 4°C to ensure all droplets settle at the bottom of the wells.

Step 2.1.3: Place the plate in a PCR machine and incubate at 65°C for 5 min. Immediately put the plate on the ice to cool for 2 min.

Step 2.1.4: Prepare the cDNA synthesis reaction mix as shown in Table 1.

**[Tip]** Two types of primers can be used in this step: Oligo(dT)_23_ VN and Random hexamers. Random hexamers are less specific than Oligo(dT)_23_ VN but may be useful for amplifying a broader range of sequences.

**Table 1:** cDNA synthesis reaction mix

Component	Per well (µL)	One plate (µL)
2× RT Mix	10	1000
Hiscript II Enzyme Mix	2	200
Oligo (dT)_23_ VN (50 μmol/L)	1	100
Total	13	1300

Step 2.1.5: Distribute 13 µL of the reaction mix into each well of the 96-well plate containing 7 µL catch buffer.

Step 2.1.6: Seal the plate with a sealing film and centrifuge the plate at 400*g* for 30 s to ensure all liquid settles at the bottom of the wells.

Step 2.1.7: Run the reverse transcription program on the PCR machine at 50°C for 45 min, then 85°C for 2 min.

**[Tip]** For random hexamers, a 25-min incubation at 25°C is required before running the above reverse transcription program. The cDNA product can either be used immediately for PCR or stored at −80°C.

#### Step 2.2: First round of PCR (amplification of IgH, IgK and IgL genes)

Step 2.2.1: Prepare the PCR premix for IgH, IgK and IgL amplification as shown in Table 2.

**Table 2:** PCR premix for IgH, IgK and IgL amplification

Component	Per well (µL)	One plate (µL)	Final concentration
Water	7.45	745	-
Phanta buffer (2×)	10	1000	-
dNTP (10 mmol/L)	0.4	40	0.2 mmol/L
1mFH/K/L mix (50 µmol/L)	0.15	15	0.38 µmol/L
1mRG/K/L (50 µmol/L)	0.1	10	0.25 µmol/L
Phanta polymerase	0.4	40	-
Total	18.5	1850	-

To prepare the 1mFH/K/L (forward primer) mix, first dilute each forward primer to 50 µmol/L, then combine 10 µL of each diluted primer in a 1.5-mL tube to make the primer mix (50 µmol/L). The 1mRG/K/L reverse primer is also diluted directly to 50 µmol/L. 15 µL of the forward primer (1mFH/K/L) mix and 10 µL of the reverse primer (1mRG/K/L) are used for a single 96-well plate.

**[Tip]** The primers used in the first PCR have been previously published (von Boehmer *et al*. 2016). For IgH and IgK amplification, two primer sets are available: one optimized for normal-yield cDNA and the other for low-yield cDNA.

Step 2.2.2: Distribute 18.5 µL of the PCR premix into each well of the 96-well PCR plate.

Step 2.2.3: Add 1.5 µL of cDNA from Step 2.1.7 to the corresponding wells.

Step 2.2.4: Seal the plate with PCR film and perform the following thermal cycling program (Table 3).

**Table 3:** Thermal cycling program for IgH, IgK and IgL amplification

Step	Temperature	Time	Cycles
Initial denaturation	95°C	30 s	1
Denaturation	95°C	15 s	50
Annealing	46°C	15 s	50
Extension	72°C	1 min	50
Final extension	72°C	10 min	1
Hold	16°C	∞	-

Step 2.2.5: The PCR product can be used immediately for the next round of PCR or stored at −20°C or −80°C.

#### Step 2.3: Second round of PCR (seq-PCR, IgH and IgK/L amplification for sequence analysis)

Step 2.3.1: Prepare the PCR premix for the second round of amplification (Table 4).

**Table 4:** PCR premix for the second round of amplification

Component	Per well (µL)	One plate(µL)	Final concentration
Water	7.64	764	-
2× Es Taq MasterMix	10	1000	-
2mFG/K or 2mFL mix (50 µmol/L)	0.18	18	0.45 µmol/L
2mRG/K/L (50 µmol/L)	0.18	18	0.45 µmol/L
Total	18	1800	-

The 2mFG and 2mFK primers (forward), and the 2mRG, 2mRK, and 2mRL primers (reverse), each consist of a single primer, whereas the 2mFL mix is prepared by combining two primers in equal amounts, using the same method as the 1mFH mix described earlier.

**[Tip]** The primers used in the second PCR (seq-PCR) have been previously published (von Boehmer *et al*. 2016).

Step 2.3.2: Distribute 18 µL of the premix into the wells of a new 96-well PCR plate.

**[Tip]** The total volume can be adjusted based on the requirements of the sequencing purpose.

Step 2.3.3: Add 2 µL of the first PCR product to the corresponding wells.

Step 2.3.4: Seal the plate with PCR film and perform the following thermal cycling program (Table 5).

Step 2.3.5: Prepare 1% agarose gel to check the PCR products. The expected product should be between 400−600 bp.

Step 2.3.6: The IgH and IgK/L PCR products with correct sizes are purified and sequenced using the reverse primers.

**Table 5:** Thermal cycling program for the second round of amplification

Step	Temperature	Time	Cycles
Initial denaturation	94°C	2 min	1
Denaturation	94°C	30 s	40
Annealing	55°C	30 s	40
Extension	72°C	20 s	40
Final extension	72°C	10 min	1
Hold	16°C	∞	-

**[Tip]** Using the reverse primers for sequencing helps ensure the identification of the J portion of the heavy chain or light chain, which is important for the cloning PCR in Step 2.4. Variable and junctional (J) gene segments can be identified using the immunoglobulin BLAST search tool available on the NCBI website (http://www.ncbi.nlm.nih.gov/igblast/). If the success rate is low, retain the same reverse primers, but try the forward primer mix used for the cloning PCR for the second PCR amplification. The results from these two sets of primers may differ, but any PCR product within the 400−600 bp range can be recovered for the next step.

#### Step 2.4: Third round of PCR (clone-PCR, variable genes amplification for expression vector cloning)

Step 2.4.1: Prepare the PCR premix for high-fidelity amplification (Table 6).

To prepare the mVH/K/L (forward primer) mix, first dilute each mVH/K/L primer to 50 µmol/L, then combine 10 µL of each diluted primer in a 1.5-mL tube to make the primer mix (50 µmol/L). Typically, the mJH/K/L (reverse) primer is selected based on the antibody sequence and only one reverse primer is required. The primers used in the third PCR are adapted from published primers and listed in Tables 7−9 (von Boehmer *et al*. 2016; Ho *et al*. 2016).

Step 2.4.2: Distribute 18.5 µL of the PCR mix into each well of a 96-well PCR plate.

**Table 6:** PCR premix for high-fidelity amplification

Component	Per well (µL)	One plate (µL)	Final concentration
Water	7.45	745	-
Phanta buffer (2×)	10	1000	-
dNTP (10 mmol/L)	0.4	40	-
mVH/K/L mix (50 µmol/L)	0.15	15	0.38 µmol/L
mJH/K/L primer (50 µmol/L)	0.1	10	0.25 µmol/L
Phanta polymerase	0.4	40	-
Total	18.5	1850	-

**Table 7:** IgH clone-PCR primers

IgH clone PCR	Primer sequence
mVH01-F	ctttttctagtagcaactgcaaccggtgtacattcccaggtgcagctgcagcagcctgg
mVH02-F	ctttttctagtagcaactgcaaccggtgtacattcccaggtgcagctgcagcagtctgg
mVH03-F	ctttttctagtagcaactgcaaccggtgtacattcccaggtgcagctgaagcagtctgg
mVH04-F	ctttttctagtagcaactgcaaccggtgtacattcccaggtgcagctgaaggagtctgg
mVH05-F	ctttttctagtagcaactgcaaccggtgtacattccgaggtgaagctggaggagtctgg
mVH06-F	ctttttctagtagcaactgcaaccggtgtacattccgaggtgcagctggtggagtctgg
mVH07-F	ctttttctagtagcaactgcaaccggtgtacattccgaagtgcagctgttggagactgg
mVH08-F	ctttttctagtagcaactgcaaccggtgtacattccgaggtgcagctgcagcagtctgg
mVH09-F	ctttttctagtagcaactgcaaccggtgtacattccgaggtgcagctgcaggagtctgg
mVH10-F	ctttttctagtagcaactgcaaccggtgtacattccgaggtgcagctgcagcagtctgt
mVH11-F	ctttttctagtagcaactgcaaccggtgtacattccgaggtgaagctggtggagtctgg
mVH12-F	ctttttctagtagcaactgcaaccggtgtacattcccagatccagctgcagcagtctgg
mVH13-F	ctttttctagtagcaactgcaaccggtgtacattcccaggttcagctgcaacagtctga
mVH14-F	ctttttctagtagcaactgcaaccggtgtacattccgagttccagctgcagcagtctgg
mVH15-F	ctttttctagtagcaactgcaaccggtgtacattccgatgtacagcttcaggagtcagg
mVH16-F	ctttttctagtagcaactgcaaccggtgtacattccgaggtgcagcttgttgagtctgg
mVH17-F	ctttttctagtagcaactgcaaccggtgtacattcccagcgtgagctgcagcagtctgg
mVH18-F	ctttttctagtagcaactgcaaccggtgtacattccgacgtgaagctggtggagtctgg
mVH19-F	ctttttctagtagcaactgcaaccggtgtacattccgaagtgatgctggtggagtctgg
mVH20-F	ctttttctagtagcaactgcaaccggtgtacattcccaggtgcagcttgtagagaccgg
mVH21-F	ctttttctagtagcaactgcaaccggtgtacattcccagatgcagcttcaggagtcagg
mVH22-F	ctttttctagtagcaactgcaaccggtgtacattcccaggcttatctacagcagtctgg
mVH23-F	ctttttctagtagcaactgcaaccggtgtacattccgagttccagctgcagcagtctgg
mJH01-R	gaagaccgatgggcccttggtcgacgctgaggagacggtgaccgtgg
mJH02-R	gaagaccgatgggcccttggtcgacgctgaggagactgtgagagtgg
mJH03-R	gaagaccgatgggcccttggtcgacgctgcagagacagtgaccagag
mJH04-R	gaagaccgatgggcccttggtcgacgctgaggagacggtgactgagg

**Table 8:** IgK clone-PCR primers

IgK clone PCR	Primer sequence
mVK01-F	ctttttctagtagcaactgcaaccggtgtacattccaacattatgatgacacagtcgcc
mVK02-F	ctttttctagtagcaactgcaaccggtgtacattccaacattgtgctgacccaatctcc
mVK03-F	ctttttctagtagcaactgcaaccggtgtacattcccaaattgttctcacccagtctcc
mVK04-F	ctttttctagtagcaactgcaaccggtgtacattcccaaattgttctctcccagtctcc
mVK05-F	ctttttctagtagcaactgcaaccggtgtacattccgaaaatgttctcacccagtctcc
mVK06-F	ctttttctagtagcaactgcaaccggtgtacattccgaaacaactgtgacccagtctcc
mVK07-F	ctttttctagtagcaactgcaaccggtgtacattccgaaattgtgctcactcagtctcc
mVK08-F	ctttttctagtagcaactgcaaccggtgtacattccgacatcaagatgacccagtctcc
mVK09-F	ctttttctagtagcaactgcaaccggtgtacattccgacatccagatgaaccagtctcc
mVK10-F	ctttttctagtagcaactgcaaccggtgtacattccgacatccagatgactcagtctcc
mVK11-F	ctttttctagtagcaactgcaaccggtgtacattccgacattgtgatgactcagtctc
mVK12-F	ctttttctagtagcaactgcaaccggtgtacattccgacattgtgatgtcacagtctcc
mVK13-F	ctttttctagtagcaactgcaaccggtgtacattccgacattgtgctgacccaatctcc
mVK14-F	ctttttctagtagcaactgcaaccggtgtacattccgatatccagatgacacagactac
mVK15-F	ctttttctagtagcaactgcaaccggtgtacattccgatgttgtgatgacccaaactcc
mVK16-F	ctttttctagtagcaactgcaaccggtgtacattccgaaatccagatgacccagtctcc
mVK17-F	ctttttctagtagcaactgcaaccggtgtacattccgacatccagatgacacaatcttc
mVK18-F	ctttttctagtagcaactgcaaccggtgtacattccgacatccagatgacccagtctcc
mVK19-F	ctttttctagtagcaactgcaaccggtgtacattccgacatcctgatgacccaatctcc
mVK20-F	ctttttctagtagcaactgcaaccggtgtacattccgacattgtgctcacccaatctcc
mVK21-F	ctttttctagtagcaactgcaaccggtgtacattccgatgttgtggtgactcaaactcc
mVK22-F	ctttttctagtagcaactgcaaccggtgtacattccaacattgtaatgacccaatctcc
mVK23-F	ctttttctagtagcaactgcaaccggtgtacattccgatgttttgatgacccaaactcc
mVK24-F	ctttttctagtagcaactgcaaccggtgtacattccgatattgtgatgactcaggctgc
mVK25-F	ctttttctagtagcaactgcaaccggtgtacattccgacatccagatgattcagtctcc
mVK26-F	ctttttctagtagcaactgcaaccggtgtacattccgacatcttgctgactcagtctcc
mVK27-F	ctttttctagtagcaactgcaaccggtgtacattccgatgtccagatgattcagtctcc
mVK28-F	ctttttctagtagcaactgcaaccggtgtacattccgatgtccagataacccagtctcc
mJK01-R	agacagatggtgcagccaccgtacgtttgatttccagcttggtg
mJK02-R	agacagatggtgcagccaccgtacgttttatttccagcttggtc
mJK04-R	agacagatggtgcagccaccgtacgttttatttccaactttgtc
mJK05-R	agacagatggtgcagccaccgtacgtttcagctccagcttggtc

**Table 9:** IgL clone-PCR primers

IgL clone PCR	Primer sequence
mVL01/2-F	ctttttctagtagcaactgcaaccggtgtacattcccaggctgttgtgactcag
mVL03-F	ctttttctagtagcaactgcaaccggtgtacattcccaacttgtgctcactcag
mJL01-R	tgttggcttgaagctcctcactcgagggcggaacagagtgaccgagggggcagc
mJL02/03-R	tgttggcttgaagctcctcactcgagggcgaaacagggtgactgatggcgaagac
mJL04/05-R	tgttggcttgaagctcctcactcgagggcgaaacacggtgagagtgggagtggac

Step 2.4.3: Add 1.5 µL of the first-round PCR product as a template to each well, ensuring corresponding IgH and IgK/L products are in their respective wells.

Step 2.4.4: Seal the plate with PCR film and run the following thermal cycling program (Table 10).

**Table 10:** Thermal cycling program for high-fidelity amplification

Step	Temperature	Time	Cycles
Initial denaturation	95°C	30 s	1
Denaturation	95°C	15 s	30
Annealing	55°C	15 s	30
Extension	72°C	30 s	30
Final extension	72°C	10 min	1
Hold	16°C	∞	-

Step 2.4.5: Prepare 1% agarose gel to check the PCR products, which should be within the range of 400 to 600 bp.

Step 2.4.6: Select the bands that correspond to the correct size for the following steps, then purify the IgH and IgK/L PCR products using a gel extraction kit.

**Figure 2:**
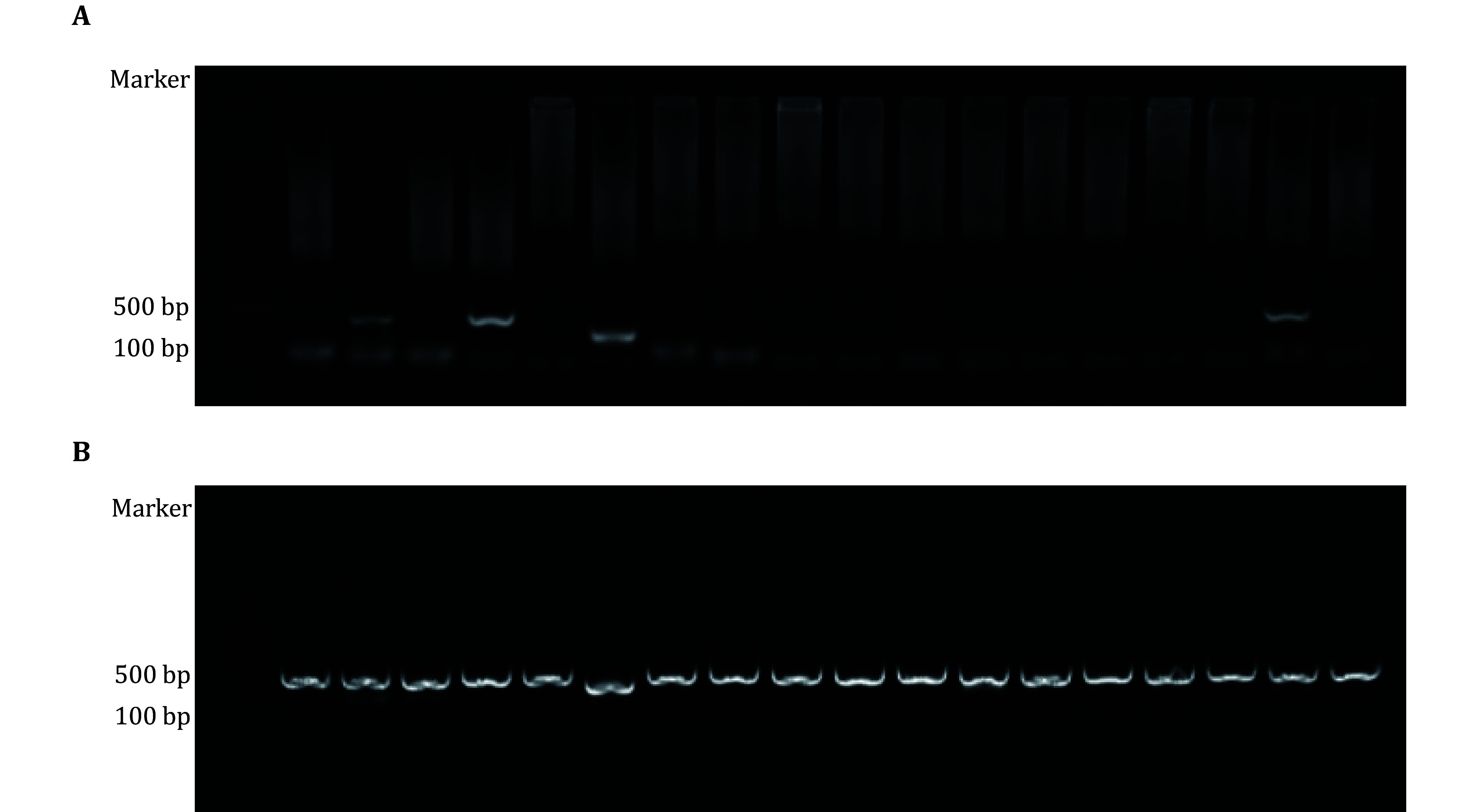
Clone-PCR comparison using templates from the first or second round of PCR. **A** Representative agarose gel image showing cloning PCR results using first-round PCR products as templates. **B** Representative agarose gel image showing cloning PCR results using second-round PCR products as templates

**[Tip]** If no bands are observed in the wells that were positive in the seq-PCR, the seq-PCR product can be utilized as a template (Fig. 2). Although the sequences at the 3’ or 5’ ends may be inaccurate, this approach allows for initial functional verification before sequence correction.

#### Step 2.5: Cloning into expression vectors

The PCR-amplified IgH and IgK/L genes are ligated into mammalian expression vectors containing the mouse IgG1 or IgK or human IgG1, IgL, or IgK constant regions, enabling mAb production.

Step 2.5.1: Prepare a DNA assembly mix (Table 11).

**Table 11:** DNA assembly mix

Component	Per well (µL)
5× CE II Buffer	1
Exnase II	0.5
Linearized vector (about 5000 bp, 20−25 ng/µL)	1
Insert segment (about 500 bp, 5 ng/µL)	1
ddH_2_O	1.5
Total	5

**[Tip]** It is recommended to first prepare the assembly mix without the gene fragment. Distribute 4 µL of the assembly mix into each well of a 96-well plate for convenience. Then, add 1 µL of the diluted insert to the corresponding wells. We typically use a molar ratio of 1:2 for vector to PCR insert, with a total reaction volume of 5 µL usually being sufficient.

Step 2.5.2: Incubate the plate at 37°C for 30 min. The assembled product can be stored at −20°C for later use or directly applied for transformation.

#### Step 2.6: Transformation and plasmid preparation

Step 2.6.1: Thaw 2 mL of competent *E. coli* cells on ice. Aliquot 20 µL of cells into each well of a 96-well plate. Adjust the volume of competent cells as necessary for the experiment.

Step 2.6.2: Add 1 µL of the assembled product to each well. Incubate the plate on ice for 30 min.

Step 2.6.3: Perform a heat shock at 42°C for 35 s, followed by a 2-min incubation on ice.

Step 2.6.4: Add 100−200 µL of LB medium to each well. Incubate the plate at 37°C with shaking at 220 r/min for 1 h.

Step 2.6.5: Plate 100 µL of the transformation culture onto LB agar plates containing the appropriate antibiotic. Incubate the plates at 37°C for 12−16 h until colonies form.

Step 2.6.6: Select two colonies for each gene and culture them overnight in an LB medium with the appropriate antibiotic for plasmid preparation.

Step 2.6.7: Extract the plasmid DNA and verify the insertion through a double enzyme restriction digestion.

### Step 3: Antibody expression and binding screening

#### Step 3.1: Small volume expression and binding verification

To minimize costs and reduce the number of large-volume expressions needed for candidate antibodies, we introduce small-volume antibody expressions prior to scaling up to medium volumes (20−200 mL). This approach strikes a balance between scalability and precision, offering an efficient and practical method for monoclonal antibody development.

Step 3.1.1: Culture Expi293F cells in a 12-well plate (non-tissue culture-treated), to a cell density of 2 × 10⁶ to 3 × 10⁶ cells/mL.

**[Tip]** A 12-well plate is used as an example. Depending on experimental requirements, 24-well or 48-well plates can also be used to adjust for varying cell culture volumes or throughput.

Step 3.1.2: Dilute 1.5 µg of plasmid DNA (IgH: IgK/L = 1:1.2) in medium without FBS and antibiotics, and prepare 2.25 µL of PEI solution per antibody, or use another transfection reagent according to the manufacturer's instructions to form the transfection mixture.

**[Tip]** Due to the small amount of plasmid required for the initial transfection, prepare plasmid DNA using a Plasmid Mini Kit, which typically yields sufficient plasmid for initial expression experiments (40−50 mL). Alternatively, use a 96 Endo-Free Plasmid Kit to obtain enough plasmid for plate-based expression (1−2 mL).

Step 3.1.3: Add the transfection mixture to each well containing Expi293F cells. Leave one well untreated or transfected with an empty vector as a control.

**[Tip]** After transfection, seal the edges of the cell plate with sealing film to prevent rapid evaporation of culture medium from edge wells, which could negatively affect cell viability and expression efficiency.

Step 3.1.4: Incubate the transfected cell plate at 37°C in a CO₂ incubator with shaking at 120 r/min. Add the expression enhancer solution during the incubation to improve expression levels.

Step 3.1.5: Collect the cell supernatants containing expressed mAbs at 3, 4, 5, or 6 days of post-transfection for initial binding screening. Supernatants from untreated or empty vector-transfected wells should serve as negative controls.

Step 3.1.6: For functional verification, perform ELISA or flow cytometry using the collected supernatant. ELISA: coat wells with the target antigen, add supernatants as the primary antibody, and use HRP-conjugated goat anti-human/mouse Fc as the secondary antibody to detect binding. Flow cytometry: after blocking, incubate antigen-expressing cells (*e*.*g*., 293T cells transfected with plasmid encoding the target antigen) with the collected supernatants. Subsequently, stain the cells with FITC-conjugated goat anti-human/mouse Fc antibodies. Antibodies exhibiting binding to the target antigen will result in FITC-positive signals, distinguishable from controls. Ensure that supernatants from untreated wells or wells transfected with an empty vector are included as negative controls to validate specificity.

#### Step 3.2: Medium-volume expression and purification

After binding screening, positive mAbs will be subjected to medium-scale expression for further analysis.

Step 3.2.1: Culture Expi293F cells in a 125 mL shake flask to a cell density of 2 × 10⁶ to 3 × 10⁶ cells/mL.

Step 3.2.2: One day before transfection, dilute the cells to a concentration of 1 × 10⁶ to 1.5 × 10⁶ cells/mL in 20 mL medium.

Step 3.2.3: On the day of transfection, dilute 30 µg of plasmid DNA (IgH: IgK/L = 1:1.2) in a medium without FBS and antibiotics. Prepare the transfection complex according to standard protocols.

Step 3.2.4: Add the transfection complex to the cell culture.

Step 3.2.5: Incubate the flask at 37°C in a CO₂ incubator with shaking at 120 r/min. Add the expression enhancer solution to boost expression levels. Culture the transfected cells for 96−144 h.

Step 3.2.6: Centrifuge the culture at 8000*g* for 30 min at 4°C to pellet the cells.

Step 3.2.7: Filter the supernatant through a 0.45-µm membrane to collect the antibody-containing supernatant.

Step 3.2.8: Prepare a Protein A column for antibody purification.

Step 3.2.9: Load the supernatant onto the column, and let the antibodies bind to the matrix.

Step 3.2.10: Wash the column with PBS containing 1 mol/L NaCl, followed by PBS alone to remove contaminants.

Step 3.2.11: Elute bound antibodies using a low-pH buffer and immediately neutralize the elution with a neutralization buffer. A recommended system is 0.1 mol/L Glycine (pH 2.7) for elution and 1 mol/L Tris-HCl (pH 9.0) for neutralization in a 5:1 volume ratio.

Step 3.2.12: Verify antibody concentration and purity via SDS-PAGE. Store purified antibodies in aliquots at −80°C for long-term storage or at 4°C for short-term use. Further functional validation can be performed using ELISA, flow cytometry or alternative experiments for antibody verification.

The optimized protocol combines a 96-well format of single-cell cloning and a 12-well format of antibody expression, offering several advantages over traditional methods in antibody development. By using FACS to select antigen-specific B cells and efficient single-cell RT-PCR, followed by high-throughput cloning and expression, our approach accelerates the entire process while minimizing the cost required for antibody discovery. Although the improvements may not be revolutionary, this protocol provides a reliable and reproducible method for mAb production in mice, complementing the current pipeline for antibody discovery, immune profiling, and applications in diagnostics and therapeutics.

## Conflict of interest

Xuanxiu Ren, Yiwei Zhang, Gan Zhang, Shangyu Yang, Feiyang Yu, Rao Cheng, Zengqin Deng and Haiyan Zhao declare that they have no conflict of interest.
